# Optimising oncology drug expenditure in Ireland

**DOI:** 10.1007/s11845-024-03672-y

**Published:** 2024-04-03

**Authors:** Ruth Kieran, Maeve Hennessy, Kate Coakley, Hazel O’Sullivan, Tim Cronin, Daire Lynch, Eibhlin Mulroe, Katie Cooke, Dearbhaile Collins, Seamus O’Reilly

**Affiliations:** 1 Department of Medical Oncology, Cork University Hospital, Cork, Ireland; 2 Cancer Research@UCC, College of Medicine & Health, University College Cork, Cork, Ireland; 3 College of Medicine & Health, University College Cork, Cork, Ireland; 4 Cancer Trials Ireland, RCSI House, 121 St Stephen’s , Dublin 2, Green, Ireland; 5 Department of Pharmacy, Cork University Hospital, Cork, Ireland

**Keywords:** Biosimilars, Clinical trials, Expanded access programmes, Personalised therapy, Pharmacoeconomics, Therapy rationalisation

## Abstract

A combination of improvements in patient survival, increasing treatment duration, and the development of more expensive agents has led to a doubling of per-capita spending on cancer medicines in Ireland (2008–2018). Despite this, access to new drugs is poor in comparison to other EU countries. We examine methods to optimise oncology drug spending to facilitate access to newer anticancer agents. Key targets for spending optimisation (biosimilar use, clinical trials and expanded access programs, waste reduction, avoidance of futile treatment, and altered drug scheduling) were identified through an exploratory analysis. A structured literature search was performed, with a focus on articles relevant to the Irish Healthcare system, supplemented by reports from statutory bodies. At the present time, EMA-approved agents are available once approved by the NCPE. Optimising drug costs occurs through guideline-based practice and biosimilar integration, the latter provides €80 million in cost savings annually. Access to novel therapies can occur via over 50 clinical trials and 28 currently available expanded access programmes. Additional strategies include reversion to weight-based immunotherapy dosing, potentially saving €400,000 per year in our centre alone, vial sharing, and optimisation of treatment schedules. A variety of techniques are being employed by oncologists to optimise costs and increase access to innovation for patients. Use of biosimilars, drug wastage, and prescribing at end of life should be audited as key performance indicators, which may lead to reflective practice on treatment planning. Such measures could further optimise oncology drug expenditure nationally facilitating approval of new agents.

## Introduction

The past two decades have been characterised by a significant expansion in new treatment options. While only one new treatment was approved annually in the 1990s, by 2020, 10 new medicines were being approved per year, with 17 new approvals in 2021 [1]. Alongside improvements in diagnostics, surgery, and supportive care, these developments have greatly improved outcomes for many of our patients, for example in women diagnosed with ER + /Her2 + de novo metastatic breast cancer, approximately 75% are still alive after 5 years, compared to under 30% of those diagnosed in the pre-trastuzumab era [2].

As patients with advanced disease live longer, treatment durations are increasing, and in addition to the high costs of some of the more newly authorised drugs (Table 1), this has led to a commensurate increase in government spending, with per-capita spending on cancer medicines in Ireland doubling from €29 to €64/capita between 2008 and 2018 [3]. Annual drug spending exceeds €300 million [3], representing 27% of state spending on cancer, and 0.1% of total GDP. Despite this, Irish patients access to new oncology drugs remains relatively poor [1], at least partly due to financial constraints. Of the new drugs approved by the European Medicines Agency (EMA) between 2017 and 2020, only 51% were reimbursed by the HSE at the beginning of 2022, placing Ireland second last among the EU-15 countries [1].

**Table 1:** Current expenditure on highest-grossing solid-tumour drugs (by sales)

Drug	Reimbursed indications (Ireland)	Global sales (2021) [4]	Monthly cost per patient^b^
Pembrolizumab^a^	• Melanoma • Advanced non-small cell lung cancer (NSCLC) • Advanced urothelial carcinoma • Advanced head and neck squamous cell carcinoma (HNSCC) • Classical Hodgkin lymphoma • Advanced cervical cancer	€17 billion	€8000
Nivolumab^a^	• Advanced melanoma • Advanced NSCL • Advanced renal cell carcinoma • Advanced HNSCC • Classical Hodgkin lymphoma	€7 billion	€7,500 [5]
Palbociclib	Hormone-sensitive Her2 negative advanced breast cancer	€5.1 billion	€2570 [6]
Pertuzumab	Her2 expressing breast cancer	€4 billion	€4000
Atezolizumab	Urothelial cancer NSCLC Small cell lung cancer Triple-negative breast cancer	€3.4 billion	€6500

^a^WHO essential medicines list

^b^Extrapolated from available data; prices being paid may be subject to confidential negotiations and may in reality be lower than shown here

Alongside advocating for further resources, it is essential that oncologists try to maximise value for money using the available resources. In this article, we will examine mechanisms to provide high-quality care while reducing drug spending, including accessing treatment via expanded access programmes and clinical trials, use of biosimilar agents, optimisation of drug scheduling and dosing, reduction of drug wastage, and avoidance of treatment which is unlikely to be beneficial, particularly at the end of life.

## Clinical trials

Clinical trials afford patients access to novel therapies and are essential in driving continued progress and advances in cancer care. Furthermore, they have clear economic benefits, as in industry-sponsored studies, the trial drugs are provided free of charge. The cost implications are significant: in a pre-pandemic review in Cork University Hospital, novel therapies were administered to patients to a value of €50,000–€90,000 each month [7]. A review of clinical trials currently open through Cancer Trials Ireland demonstrates 36 solid-tumour studies offering patients access to investigational agents (Table 2), with an additional 14 haematology trials open. Of the 287 patients accrued to these studies in 2022, 137 (48%) participated in trials investigating immunotherapies, including some of the most expensive marketed drugs. A previous analysis estimated that the return from state investment into cancer trials in Ireland is twice that which is invested, representing a significant return on investment to the state [8]. In 2016 the combined €3.63m funding from the Exchequer and other grants resulted in a saving of an estimated €6.5m in the cost of cancer drugs. Additionally, nearly €6m in tax revenues was generated with a contribution of €16.5m to Ireland’s gross domestic product (GDP) as well as support for over 230 largely highly skilled jobs [7]. One clinical trials unit included in this analysis reported that each €1 in grant funding received, yielded €3 in income from industry for trials, supporting the need for continued expansion and investment in cancer research units nationwide. In international studies, average drug cost avoidance per patient has been estimated at €10,000 to €16,000 [9, 10], with cost savings per centre up to €6 million a year [11], significantly exceeding the costs associated with running a clinical trial [12].

**Table 2:** Studies with investigational agent arm currently accruing adult patients via Cancer Trials Ireland (April, 2023, not including haematology trials)

	Trial	Investigational arm	Comparator arm	Irish recruitment target
Breast cancer				
1.	EPIK-B5	Alpelisib and Fulvestrant	Placebo and fulvestrant	12
2.	KEYNOTE-B49	Pembrolizumab and therapy of physician choice (TPC)	TPC	Not specified (NS)
3.	lidERA Breast Cancer/ GO42784/ TRIO 045	Giredestrant ± LHRH agonist	Endocrine TPC ± LHRH agonist	40
4.	SASCIA	Sacituzumab govitecan	TPC	40
5.	DESTINY-Breast12	Trastuzumab deruxtecan (compares patients with/without brain metastases)		13
6.	DESTINY-Breast05	Trastuzumab deruxtecan	Trastuzumab emtansine (T-DM1)	5
7.	ZEST/ GSK 213831	Niraparib	Placebo	6
8.	ALEXANDRA/IMpassion030	Chemotherapy with concomitant and maintenance atezolizumab	Chemotherapy	28
Lung cancer				
9.	Krystal-7	Adagrasib and pembrolizumab	Platinum and pemetrexed and pembrolizumab	4
10.	MK7684A-008	Pembrolizumab/vibostolimab co-formulation and platinum/etoposide	Atezolizumab and platinum/etoposide	6 +
11.	KRYSTAL-12	Adagrasib	Docetaxel	9
12.	CA224-104 (RELATIVITY)	Relatlimab and nivolumab and chemotherapy	Nivolumab and chemotherapy	6
13.	AcceleRET-Lung	Pralsetinib	TPC	3
14.	AbbVie M14-239	Telisotuzumab vedotin (assessing response rate)		9
Skin cancer				
15.	MK-3475–630/KEYNOTE-630	Pembrolizumab	Placebo	4–8
16.	R2810-ONC-1788	Cemiplimab	Placebo	8
Genitourinary cancer				
17.	MK6482-012	Pembrolizumab and belzutifan and lenvatinib	Pembrolizumab and lenvatinib	7
18.	MK3475-365	Pembrolizumab combination therapies	Multiple arms	4
19.	MK6482-011	Belzutifan and lenvatinib	Cabozantinib	8
20.	MK6482-022	Belzutifan and pembrolizumab	Placebo and pembrolizumab	15
21.	IMvigor011 B042843	Atezolizumab	Placebo	9
22.	DASL HiCaP	Darolutamide and androgen deprivation therapy and radiation (ADT/RT)	Placebo and ADT/RT	45
23.	MK3475-905/ KEYNOTE-905/ EV-303	Perioperative enfortumab vedotin and pembrolizumab	Cystectomy alone	8
Head and neck cancer				
24.	CompARE	Cisplatin and radiation and durvalumab	Cisplatin and radiation	7
Gastrointestinal				
25.	LEAP-015 (MK-7902–015)	lenvatinib plus pembrolizumab plus chemotherapy	Chemo alone	20
26.	DESTINY DS8201-A-U306	Trastuzumab deruxtecan	Ramucirumab and paclitaxel	8–9
27.	Astellas 8951-CL-5201	Zolbetuximab, chemotherapy	Chemotherapy	3
28.	Lithium	Lithium, chemotherapy (dose escalation study)		24
29.	ZWI-ZW25-301	Zanidatamab/chemotherapy ± tislelizumab	Trastuzumab/chemotherapy	6
Gynaecological cancers				
30.	innovaTV 301 / ENGOT cx12	Tisotumab vedotin	TPC chemotherapy	20
31.	ENGOT-en15/ KEYNOTE-C93-00/ GOG-3064	Pembrolizumab	Platinum/paclitaxel	6
32.	ENGOT-ov65 / KEYNOTE-B96	Pembrolizumab plus paclitaxel ± bevacizumab	Placebo and paclitaxel ± bevacizumab	NS
33.	OVIHIPEC-2	Surgery with HIPEC	Surgery alone	NS
Basket trials (multiple/unknown tumour type)				
34.	CUPISCO	Targeted/immunotherapy guided by NGS	Platinum	NS
35.	LOXO 101	LOXO 101 (assess response rate)		7
36.	LYNK-002	Olaparib (assess response rate)		NS

Fewer than 5% of patients participate in clinical trials globally, and Irish data is consistent with this [13, 14]. Where trials are offered to patients, fewer than 5% of patients decline participation [13, 14], and the major barrier remains a lack of suitable open trials for tumour type/stage within a reasonable travel distance for the patient.

## Expanded access programmes

Expanded access programmes (EAPs) allow patients to access new drugs outside of clinical trials [15], and may be utilised if a drug is approved by regulatory bodies but not yet reimbursed by the state [16]. A common model for these programmes is that the programme sponsor (typically the pharmaceutical company that hopes to market a new drug) provides the drug for free to selected patients after an application by their treating consultant; however, the programme may close to new patients at any time, sometimes before reimbursement is agreed. In Ireland, unlike most other European countries, there is no national protocol or standardised approach for the implementation of EAPs [17]. Consequently, there is a gap in the literature in terms of data and research examining the role that these programmes play in current cancer patient care in Ireland. Cronin et al. [18] assessed the role of expanded access medications in 3 oncology units. Over a 9-year period 193 patients had been enrolled in programmes. Importantly, the results also show that the number of EAPs being accessed increased annually, emphasising the increasing gap between drug approval and drug reimbursement in this jurisdiction. The Irish Society of Medical Oncology (ISMO) has established a registry of expanded access programmes to ensure equity of access of patients. A total of 33 drugs are currently provided for a total of 50 different cancer indications, reflecting the important role of EAPs in the landscape of cancer practice nationally (Table 3).

**Table 3:** Expanded access programmes currently open to patients in Ireland with solid tumour malignancies, April, 2023

	Programme	Indication	Contact
GI cancer	Durvalumab	Locally advanced/metastatic bilary tract cancer, in combination with chemotherapy	AstraZeneca
	Liposomal Irinotecan	Metastatic adenocarcinoma of the pancreas following progression on gemcitabine-based therapy	Servier Laboratories
	Encorafenib	BRAF V600E mutant metastatic colorectal cancer, with cetuximab	Pierre Fabre
	Regorafenib	Hepatocellular carcinoma, previously treated with sorafenib	Bayer
	Atezolizumab	Advanced/unresectable hepatocellular carcinoma, with bevacizumab	Roche
Lung cancer	Amivantamab	Advanced NSCLC with activating EGFR exon 20 insertion mutations	Janssen
	Atezolizumab	Adjuvant NSCLC, PDL1 ≥ 50%, post 4 cycles of platinum-based chemotherapy	Roche
	Dacomitinib	Locally advanced/metastatic NSCLC with EGFR mutations	Pfizer
Gynaecological cancers	Dostarlimab	Post platinum, recurrent dMMR endometrial cancer	GSK
	Lenvatinib	Relapsed/recurrent pMMR endometrial cancer, with pembrolizumab	Eisai
Breast cancer	Trastuzumab-deruxtecan	Unresectable/metastatic HER2-positive breast cancer, post 2 + prior anti-HER2-based regimens	AstraZeneca/ Daiichi-Sankyo
	Abemaciclib	ER + , HER2- metastatic breast cancer	Eli Lilly
	Alpelisib	PIK3CA-related overgrowth syndrome	Novartis
Genitourinary	Avelumab	Advanced/metastatic urothelial cancer, post-platinum	Merck-Pfizer
	Darolutamide	Non-NCCP Protocol Approved Prostate Cancer	Bayer
	Enfortumab vedotin	Locally advanced or metastatic urothelial cancer, previous platinum and previous PD1/PD-L1 inhibitor	Astellas
Thyroid/head and neck	Sorafenib	Progressive, locally advanced/metastatic differentiated thyroid carcinoma, refractory to radioactive iodine	Bayer
	Selpercatinib	Patients under 18 years with RET-altered thyroid cancers	Eli Lilly
Melanoma	Tebentafusp	Uveal melanoma	Immunocore
Other/multiple indications	Larotrectinib	Locally advanced/unresectable tumours with NTRK gene fusion, or where surgical resection is likely to result in severe morbidity and have no satisfactory treatment options	Bayer
	Erdafitinib	Metastatic carcinoma with confirmed FGFR alteration, having exhausted standard of care therapies	Janssen
	Entrectinib	1. NTRK fusion-positive solid tumour 2. ROS-1 positive NSCLC	Roche
	Adagrasib	KRAS G12C mutant tumours	Mirati Therapeutics
	Pembrolizumab	1. Metastatic/ Neoadjuvant/Adjuvant TNBC 2. Relapsed/recurrent pMMR endometrial cancer, with Lenvatinib	MSD
	Neratinib	Neratinib plus Trastuzumab plus Fulvestrant	Pierre Fabre

Details per ISMO list 2023—list is non-exhaustive, eligibility should be clarified with the sponsor on a per-patient basis

## Biosimilars

The term biosimilar describes a biologic drug which has been developed such that there are no ‘clinically meaningful differences’ between it and the reference medicine which has already been licenced [19]. Although the EU considers EMA licensed biosimilars interchangeable [20], unlike generic drugs, the Health (Pricing and Supply of Medical Goods) Act 2013 in Ireland specifically excludes biosimilars from being added to the ‘list of interchangeable medicinal products’, so that these medicines cannot undergo generic substitution. Because of this, clinicians must intentionally decide to switch to these, if appropriate.

To provide an idea of the scope of the cost savings provided by biosimilars, a 2019 study modelled the impact of choosing biosimilar trastuzumab (a monoclonal antibody targeting HER2) instead of its reference product (Herceptin) in 28 European countries over 5 years [21], determining that €1.13–€2.27 billion could be saved based on epidemiological data [21]. The same model estimated that 55,082–116,196 additional patients could gain access to trastuzumab over the 5-year period [21]. Real-world data has revealed savings of GBP £100 million for trastuzumab and adalimumab biosimilars the UK National Health Service (NHS) in 2018 alone [22].

Across all biosimilars, annual savings in Ireland exceed €80 million [23]. Biosimilars currently account for 52–54% of the market share of oncology biologicals in Ireland [23, 24], compared to over 90% in Austria, the Netherlands, and Norway, suggesting scope for further expansion [24].

A 2021 literature review investigated the impact of non-medical switching policies which replace reference biologic products with biosimilars [25], and found that cost savings were achieved in most cases [25], but the concept of biosimilar substitution does not currently have sufficient support from prescribers [26]. Some barriers to more extensive uptake globally identified include patient and prescriber perception of biosimilars, lack of awareness, and issues regarding interchangeability and substitution of biosimilars [27]. Recently updated American Society of Clinical Oncology (ASCO) guidelines support interchangeable use of biologics, and note that to date, no immunogenicity concerns for any approved oncology biosimilars have been identified by the FDA [28]. As experience with using biosimilars has grown substantially, hesitation from oncologists may reduce over time [22]. It has been recommended that there should be scheduled evaluations of patient experience included in biosimilar implementation plans [29], which may help strengthen the case for increased use.

## Drug dosing and scheduling

Historically, drug dosing has been determined by the maximum tolerated dose established in phase 1 trials; however, moving to a minimum efficacious dose may be more appropriate. A number of drugs are commonly used at a lower dose than that in their original trials [30], such as the PARP inhibitor niraparib, where the use of a 200 mg starting dose in patients weighing < 77kg is associated with better tolerance and can save €2700 compared to a 300 mg dose [6], without impact on survival outcomes [31].

While immunotherapies have improved outcomes for many patients, controversy exists around the 200/400 mg (pembrolizumab) or 240/480 mg (nivolumab) ‘flat’ doses that are typically prescribed, irrespective of body surface area. While early studies with pembrolizumab investigated a 2 mg/kg dose [32–34], when pharmacokinetic data suggested a 200 mg flat dose provided similar exposure distributions [35], research shifted to using this as a standard. As the median patient weight in this study was 77.2 kg, most patients received more product via flat-dose dispensing. In one Cork University Hospital study [36], flat dosing resulted in increased costs of €22,693 and €4018 per patient (pembrolizumab and nivolumab, respectively) in comparison to weight based, with an annual total cost of almost €400,000 in our hospital alone. Current product licences [37, 38], National Cancer Control Programme (NCCP) protocols [39], and most recent studies use flat-based dosing. Consequently, weight-based dosing is an off-label use; however, a global reversion to use of weight-based pembrolizumab dosing could save $0.8 billion per year [40].

Chemotherapy de-escalation in metastatic colorectal cancer is well established, where patients may receive 8–24 weeks of ‘induction’ chemotherapy with a fluoropyrimidine and oxaliplatin/irinotecan before moving to single-agent maintenance. While oxaliplatin and irinotecan are not expensive drugs by the standards of immunotherapies, no survival advantage has been seen by continuing them in meta-analysis [41]. Similarly, ‘treatment holidays’ can improve quality of life and tolerance of treatment in those with stable/responding advanced disease, particularly in colorectal cancer. While the COIN study (observation and reintroduction of therapy as-needed compared to maintenance therapy) did not meet non-inferiority endpoints [42], a later meta-analysis found no evidence of impaired survival [43], with similarly positive findings in the FOCUS4-N trial [44], while using cetuximab on an observation/reintroduction schedule saves over £35,000 per patient annually [45, 46] (almost €49,000, adjusting for inflation). In addition to savings on drug products, drug de-escalation and treatment holidays reduce demands for slots on oncology daywards, a resource which is severely limited in many hospitals [47], and which cannot easily be increased without major investment. Unlike other countries [48], Ireland does not mandate stopping immunotherapy after any set period [39]. The optimal duration of immunotherapy in metastatic disease is being investigated in trials such as DANTE [49], Safe Stop, and STOP-GAP [50], and if strong evidence is found for treatment interruptions after 2 years, this may be a significant cost reduction.

Some agents have interactions with food, which could potentially be leveraged to reduce drug costs. Abiraterone (a hormonal agent used in advanced prostate cancer) is typically taken as a 1000 mg dose on an empty stomach. A 250 mg dose taken with a high-fat meal may yield similar control of PSA [51] to the conventional 1000 mg dose, though long-term survival outcomes have not been reported. This strategy is being used in some low-income countries [52], and the US National Comprehensive Cancer Network (NCCN) guidelines include 250 mg with food as an option for patients where financial toxicity limits access to care [53]. This is not being practised in Irish centres, and a 250 mg dose is not available in the Irish market. Coadministration of grapefruit with the kinase inhibitor lapatinib could also reduce the dose required [54–56], potentially saving over €2000 per patient per month [6]. Similar avenues are being explored with other agents [57, 58], though no research on clinical outcomes has been conducted; thus, these strategies are not established enough to use in our patients.

## Avoidance of futile treatments

Preventing the use of futile treatments in palliative patients is another method of reducing the financial impact of cancer care. The receipt of systemic anticancer therapy (SACT) at the end of life is associated with poor quality of death, death in hospital, and higher health care costs [59, 60]. Consequently, 30-day mortality rate after SACT is used as a key performance indicator (KPI), both in Ireland [61] and internationally [62]. The National Cancer Strategy (2017–2026) recommends that < 25% of patients should receive SACT in the last month of life [63]. Early referrals to palliative care are associated with less aggressive end of life care [64], improved overall survival, and quality of life [65].

A retrospective study of 582 patients with advanced malignancies that underwent treatment at two Dublin hospitals between 2015 and 2017 found that 22% and 6% received SACT in the last 4 and 2 weeks of life, respectively [66]. A Cork and Mercy University Hospital study assessing immune checkpoint inhibitors (ICI) demonstrated 20% of metastatic patients prescribed ICI received this in the last 30 days of life [67]. In both studies the institutions were compliant with the Cancer Strategy’s KPI and the majority of patients (92% [67] and 88% [66]) were referred to palliative care. The timing of palliative care referrals is important, with O’Sullivan et al. demonstrating that late palliative care referrals (≤ 3 months before death) were associated with hospitalisations in the last month of life [67]. Adopting early palliative care referrals allows patients and their caregivers time to manage treatment expectations, plan end-of-life care, and ultimately reduce healthcare costs [64, 68, 69].

In addition to overtreatment in the advanced setting, efforts must be made to de-escalate unnecessary treatments in patients undergoing radical intent therapy. In breast cancer, trials have sought to reduce the number of agents given adjuvantly in HER2-positive breast cancer. Biomarkers can be used to guide adjuvant SACT selection: in early ER-positive breast cancer, the use of a somatic 21 gene recurrence score led to a 62.5% reduction in chemotherapy in Irish patients, saving the health system €1,900,000 over 8 years. In other solid tumours, circulating tumour DNA (ctDNA) is being investigated as a de-escalation tool. In the dynamic study, stage II colorectal cancer patients that were ctDNA negative postoperatively had adjuvant chemotherapy omitted, with no impact on the primary endpoint of 2-year recurrence-free survival [70]. This technology does not yet have the same sensitivity for adjuvant treatment selection in other cancers, such as NSCLC, where atezolizumab still provides benefit in ctDNA-negative patients [71].

## Reducing drug wastage

Apart from strategies to look at reducing spending, we must try to ensure that the money we do spend is used economically. Drug wastage accounts for 4–18% of all cancer drug spending in the USA [72–74], and while some of this is unavoidable, drug waste mitigation strategies have been shown to reduce drug spending by up to 17% [74]. Given the €300 million annual spending, even a small reduction in wastage is likely to be very impactful.

### Reducing wastage within the hospital

Many drugs are dispensed as single-patient vials; however, these often contain more product than a typical patient needs, with up to 33% of product wasted in some cases [73]. Guidelines in many countries advise that once opened, vials must be disposed of within 6 hours [75], but even within these constraints, saving the ‘waste’ product from two or more vials to later formulate a dose for an extra patient (‘vial sharing’) can result in significant savings. With the addition of steps such as dose banding (already standard NCCP policy), or grouping patients being treated with the same drugs on the same days, up to 45% of drug waste expenditure can be avoided [76]. Closed-system drug transfer devices used in compounding may maintain drug sterility beyond the 6-hour window [77], allowing significantly more vial sharing [78], but were not yet universal in Irish hospitals at the time of last audit [79].

We have already discussed above how the adoption of flat dosing may not be beneficial—while this practice does reduce ‘wastage’ in theory, in reality, it moves us from merely discarding leftover drug to infusing leftover drug into patients.

### Reducing wastage of oral treatments

For patients who receive oral anti-cancer therapy, drug wastage is experienced by 25–41% of patients [80–83], and in those who discontinue treatment unexpectedly, 46–85% [83, 84] have ‘leftover’ doses. The cost of these drugs can be significant, with unopened packages worth a median of €2600 per patient in a Dutch study [83]*.*

Traditionally, such medications are discarded; however, several drug repositories are now established, primarily in the USA, such as the Wexner Medical Center Oral Oncology Drug Repository Program [85], and have been endorsed by ASCO guidelines [86]. These allow patients to donate leftover medications, with the intent of these being used for un- or underinsured patients, and one European scheme found a 68% waste reduction, with net savings of $1591 in selected cohorts [87]. In non-oncological studies, most patients indicate willingness to accept re-dispensed medications [88, 89], while in a small oncology study, theoretical willingness to participate in drug reuse was over 80% [90]. To our knowledge, no adverse outcomes have been reported from existing repository programmes.

In patients at higher risks of toxicity, consideration could be given to how medications are dispensed, both by modifying the dosage formulations administered and by split-fill dispensing, where patients may collect only part of an oral cycle at a time. This keeps residual medicines within the pharmacy supply chain and allows drugs to be reallocated to other patients. Wastage via these mechanisms can be reduced significantly [91] in up to 34% of patients [81], saving $1000 [80, 81] per patient. This may be particularly important with more expensive oral agents, such as palbociclib, in which at least 18% of women require a dose reduction [92], with an estimated cost of approximately $5000 per patient [92–94]. Anticipating the potential for dose reductions when deciding how the prescription should be formulated may be another cost saver in some cases (see Table 4). While these methods are slightly more inconvenient for the patient, their economic advantages are worth considering, especially in the initial weeks of treatment. Unlike other strategies, these methods can easily be implemented by an individual clinician without significant administrative hurdles.

**Table 4:** Reducing waste by modified dispensing

Many drugs are marketed in multiple drug strengths, such as sunitinib, typically prescribed at a 50 mg daily dose. This is available in Ireland in 50-mg (€4832/28 capsules), 25-mg (€2421/28 capsules), and 12.5-mg (€1214/28 capsules) formulations [6]		
*Typical prescribing:* John starts sunitinib for the first time and receives a 28-day supply of a single 50 mg capsule daily. He has significant toxicity, and after 12 days his dose is reduced to 37.5mg. He has 16 days of leftover drug, worth €2761, which is discarded	*Split-fill prescribing:* John is asked to collect only a 14-day supply of 50mg capsules at first. When he becomes unwell on day 12, he has 2 capsules left, worth €345	*Dose-reduction anticipation*: John receives 4 × 12.5 mg capsules every day When his dose is reduced to 37.5 mg, he takes 3 × 12.5 mg capsules, and has no wasted drug

## Discussion

We have outlined potential strategies for optimising drug spending without impacting patient outcomes (Table 5). While some of these will require an initial administrative or infrastructural investment, such as altering pharmacy compounding procedures or opening local access to new clinical trials, many others can be undertaken by an individual clinician almost immediately, such as investigating options for early access drugs, moving to split fill dispensing, or de-escalating individual treatments. Though the individual impact of these simpler interventions is smaller, the cumulative effect if all staff are mindful of costs is likely to be very significant.

**Table 5:** Key strategies for optimising drug expenditure

	Summary	Potential impact	Tasks for further implementation	
			Government	Clinicians
Avoiding unnecessary treatment	Includes personalising adjuvant therapies and considering treatment holidays/therapy de-escalation, or offering best supportive care as a valid option where appropriate. May reduce the burden of time-consuming and potentially toxic treatments for patients, the use of resources such as day ward treatment slots, and drug spending.	Personalised adjuvant therapy in breast cancer saved €1,900,000 over an 8 year period Greater use of treatment holidays from agents such as cetuximab could save up to €49,000 per patient	Continue work within national cancer strategy framework to avoid chemotherapy at the end of life	• Ongoing investigator-originated studies are needed to establish safety of therapy de-escalation • Consider discussing treatment breaks with patients where clinically appropriate
Reducing drug waste	Strategies such as vial sharing, dose banding and closed system drug transfer devices can reduce wastage of intravenous drug products. Split fill dispensing and drug re-dispensing could reduce wastage for oral products.	Drug waste mitigation strategies can reduce drug spending by up to 17% internationally. Difficult to quantify in Irish context due to lack of national data, but likely to be significant given the €300 million annual drugs spending.	• Consider including drug wastage as a KPI in next national cancer strategy/data collection at national level regarding this • National level review of the viability of drug re-dispensing	• Consider trial of split fill dispensing in individual units • Awareness of which medications contribute to wastage most (high-cost/short shelf life)
Utilising biosimilars	Where biosimilar agents are available, transition to routine use of these can significantly reduce costs, both directly and by reducing the cost of the reference product.	Across all biosimilars, annual savings exceed €80 million.	Continue price negotiations where biosimilars are available	Consider switch to use of biosimilar products where more cost effective
Clinical trials	In addition to gaining access to new drugs, significant financial burden moves from the HSE to the study sponsor, who may fund staff costs, radiology or laboratory costs, and drug product. Increasing the number of open trials, and the numbers of patients enrolled, may result in greater savings.	Cost savings of over €10,000 per patient enrolled, €16.5 million overall contribution to GDP (2016 data)	• Increasing state funding for Cancer Trials Ireland • Ongoing investment in infrastructure needed to support trials (radiology, dayward) to make sites attractive to sponsors	• Ongoing awareness of open trials, willingness to refer patients to other centres for enrolment where appropriate • Continue to liaise with potential sponsors and complete study feasibility assessments
Expanded access programmes	Where drugs are EMEA approved but not yet reimbursed in Ireland, some companies may offer an Expanded Access Programme. These programmes are not publicly advertised, and ISMO has begun maintaining a database of open programmes.	Difficult to quantify due to lack of data on utilisation	• NCCP-coordinated register of availability and utilisation of EAPs	• Awareness of open programmes • Consider approaching pharmaceutical companies to enquire about options in individual cases.

At the same time, some of the cost optimisations discussed here require local or national policy changes, and significant investment of time and effort from oncology physicians, pharmacists, and other staff. While reducing spending in one area will increase funding available in other areas, and hence indirectly increase access to new drugs or treatments, oncology funding is not ringfenced, and it is possible that ‘savings’ from the oncology sector may be reallocated to an entirely different area of government spending. Allowing cost savings to be reinvested locally would require significant policy change. One precedent for this is the ‘Best Value Biologic’ initiative, used in rheumatology for drugs such as adalimumab, in which the HSE Medicines Management Programme issued information for prescribers on the selected preferred biosimilars [95], and provided a €500 gain share to the hospital for each patient switched to a preferred biosimilar [95], saving €22.7 million in a year [95]. After national rollout of NCIS, a central NCCP-led review of biosimilar usage patterns could be conducted to estimate potential cost savings of further biosimilar use and the impact of a similar incentive strategy in oncology. If successful, this could be expanded to other areas of cost optimisation; however, it would require a HSE and hospital commitment that incentive funding was provided in addition to routine funding increases, not instead of.

The current (2017–2026) Cancer Strategy [63] aimed to increase participation in therapeutic clinical trials to 6% by 2020. Updated figures were not available as of 2021 [61], and the NCCP aims to improve intelligence gathering via the National Cancer Information System after its national implementation. Monitoring of this KPI is essential, and we must continue investing in our hospitals, investigators, and trials units, to make Irish study sites maximally attractive to sponsors, as well as develop additional non-industry sponsored trials. In addition to trials investigating new agents, increased funding for investigator-initiated trials could focus on dose and scheduling optimisation. The ASCO Value in Cancer Care consortium is continuing to explore avenues such as leveraging drug interactions to achieve cost savings [96], and this is an evolving research area that may yield more options in the future.

Although an informal database of the expanded access programmes currently open has been created, this is maintained by ISMO volunteers, and no information on activity or patient outcomes is available, except to the programme sponsor. A national database for all expanded access programmes would be desirable, with mandatory registration by prescribers. This would provide real-world data on activity and toxicity, often in rare tumour subtypes, facilitate regulatory bodies in approval assessments, and would also ensure equity of access for patients. Ideally, this would be linked with the National Cancer Information System (NCIS), a single national computerised system that will record and store information of cancer diagnosis and treatments for all patients treated within the public system. First planned in 2013, this system is slowly being expanded, but does not yet capture data from all sites, limiting our ability to capture data on other KPIs, such as those related to chemotherapy in the last month of life [61]. In the interim, individual cancer units should audit this themselves, alongside auditing rationalisation of other medications.

‘Drug wastage’ is not being captured at a national level in Ireland, and this could be considered for inclusion in the next National Cancer Strategy. While some wastage is inevitable to allow efficient patient flow through the dayward (e.g. many units begin compounding low-cost drugs before all blood results are finalised for patients who appear well, to minimise waiting times), at a hospital level, residual drug contents in vials, and dose reductions/treatment cancellations after drugs have been compounded should be monitored, and investment could be considered in closed-system transfer devices where not already in place. Research on modelling potential savings with split-fill prescribing, and into the acceptability of oncology drug repositories to Irish patients and clinicians is ongoing in Cork, and could be expanded nationally. The establishment of drug repositories required local legislative changes in many US states [97], and this is also likely to be necessary prior to the introduction of any schemes in Ireland.

Ireland is not unique in the challenges it faces, and Cortes et al. [98] have examined the global difficulties in accessing cancer medicines, including challenges that we are fortunate not to face, such as the marketing of substandard generics with limited regulatory oversight. They propose some of strategies that could be considered at an Irish or EU level, such as risk-sharing approaches or value-based pricing, or removing sales tax from all medicines. Oral medications are already VAT exempt; however, injectable products are not, which may increase financial toxicity and limit access for patients not covered by the drug payment scheme.

Ultimately while the methods discussed here may help optimise spending, as clinicians we must continue to advocate for appropriate allocation of budgetary resources, and remain mindful of what we prescribe, who we prescribe to, and when to stop prescribing.
